# The increased trend of non-drinking alcohol among adolescents: what role do internet activities have?

**DOI:** 10.1093/eurpub/cky168

**Published:** 2018-08-29

**Authors:** Peter Larm, Jonas Raninen, Cecilia Åslund, Johan Svensson, Kent W Nilsson

**Affiliations:** 1 Centre for Clinical Research, Uppsala University, Västmanland County Hospital Västerås, Uppsala, Sweden; 2 Department of Analysis and Method, The Swedish Council for Information on Alcohol and Other Drugs (CAN), Stockholm, Sweden; 3 Department of Clinical Neuroscience, Karolinska Institutet, Solna, Sweden

## Abstract

**Background:**

Recently, an increased trend toward non-drinking among adolescents has been observed in several countries. The aim of the present study is to evaluate a common suggestion in literature, that adolescents do not drink alcohol because they spend more time on the internet, monitored at home, by examining associations between internet activities (social media/chatting and computer gaming) and non-drinking.

**Methods:**

A health questionnaire was distributed to all 9th graders (15–16 years) in a mid-sized Swedish county in 2008, 2010 and 2012. In total, 7089 students returned the questionnaire.

**Results:**

In contrast to the suggestion, no association was found between total time spent on computers and non-drinking. Social media/chatting was robustly associated with a decreased probability of non-drinking across the three survey years. On the other hand, computer gaming during weekends only (OR = 1.74, CI = 1.13–2.69) or both on weekdays and weekends increased the probability of non-drinking (OR = 1.82, CI = 1.31–2.54) in 2012 only. However, neither social media/chatting nor computer gaming was associated with the increased trend of non-drinking from 2008 to 2012.

**Conclusions:**

Internet activities were in general not associated with non-drinking among adolescents aged 15–16 years in Sweden. Although, a weak positive association between computer gaming and non-drinking was found in 2012, this effect benefited the vast majority of the boys. The larger alcohol use among those with extensive social media use/chatting may indicate that these online platforms are arenas where adolescents are exposed for positive alcohol preferences and alcohol advertising without parental supervision.

## Introduction

The proportion of non-drinking adolescents has, in the past decade, increased in many European countries,^1^^,^^2^ the USA^1^^,^^3^ and in Australia.^4^ In Sweden, the proportion of non-drinking 9th graders (15–16 years) more than doubled from 15–20% in the 1990s to 60% in 2017.^5^ The increased trend of non-drinkers has occurred despite membership in European Union in 1995 having liberalized the Swedish alcohol policy, with a reduced state monopoly^6^ and increased quotas for private import,^7^ which has likely increased the availability of alcohol for adolescents. Further, the increased trend toward non-drinking among adolescents seems unrelated to socio-economic or demographic factors.^4^^,^^7^ Consequently, sociological, psychological and public health explanations have been suggested. One, preventions and health promotion interventions in schools and communities has reduced the attractiveness of alcohol for adolescents.^8^ Two, the trend is the consequence of more restrictive parental attitudes regarding approval and supply of alcohol.^7^^,^^8^ Three, the increased rates of non-drinking are due to a shift in adolescents’ leisure activities toward an increased use of the internet, in particular social media and computer games. However, few empirical studies have addressed these explanations.^8^

The present study addresses the third hypothesis and in particular the association between internet activities and non-drinking among adolescents. The suggested mechanism is that adolescents do not drink alcohol because they spend more time on the internet in domestic environments that are more easily monitored by parents.^7^ Previous studies have shown that the use of social media, in particular those displaying alcohol references, is associated with increased alcohol consumption among adolescents and college students.^9^^,^^10^ Other studies on computer gaming have yielded inconclusive findings. A large study of a Dutch population-based sample of 8478 adolescents showed no difference in the distribution of drinkers between computer gamers and non-gamers,^11^ whereas another population-based study of 1423 French adolescents aged 11–17 years found that alcohol use was more common among computer gamers than among non-gamers.^12^ A positive association between frequent computer gaming and non-drinking was found among 6000 adolescents aged 13–17 years in four European countries (Spain, the UK, Portugal and Sweden) that remained for adolescents in Spain and the UK when adjusted for covariates.^13^ Further, a large study of 21 170 students in 8th and 10th grade in the USA showed a positive association between total internet time and binge drinking.^14^ However, the latter study did not distinguish between different internet activities, whereas the other studies did not include other activities on the internet. To our knowledge, only one study has included multiple internet activities. This study was performed in a sample of 200 adolescents aged 13–17 years in the USA. Lifetime drinkers had an increased use of social media as compared with non-drinkers, whereas no difference in computer gaming was found between drinkers and non-drinkers.^15^ This study, however, was limited by a small sample size and that participants were recruited online, which biased the generalizability of the study.

The aim of the present study is to examine whether different internet activities, including total time spent on computers, social media use and computer gaming, are associated with non-drinking in a large population-based sample of 7089 Swedish 9th graders (15–16 years). Further, differences in these associations between the years 2008 and 2012 and between boys and girls are evaluated.

## Methods

### Participants

Data from the Survey of Adolescent Life in Vestmanland (SALVe) were used. This health survey is distributed biannually to all students in 9th grade (15–16 years) in the county of Västmanland, a mid-sized county in Sweden. This study used data from the surveys distributed in 2008, 2010 and 2012, since these surveys included detailed questions about computer and internet habits. In total, 2605 (50.4% girls) responded in 2008, 2439 (51.7% girls) responded in 2010 and 2045 (49.7% girls) responded in 2012. Missing data were attributed to participants who were absent, failed to return the questionnaire or failed to state their sex.

### Procedure

All students in 9th grade in the county of Västmanland were asked to complete the questionnaire during class hours. Participation was anonymous and voluntary. The present study followed the Swedish guidelines of social science and humanities, in accordance with the Declaration of Helsinki. Under Swedish law (Ethical Review Act 2003: 460), this type of anonymous study does not require ethical approval by the medical faculty. However, an advisory statement from the regional ethical board of Uppsala was requested for the surveys distributed in 2006 and 2014. The surveys were approved at both occasions.

### Measures

Covariates included country of birth, family substance problems, conflicts between parents, physical abuse, number of friends, status among friends, psychosomatic problems, sleeping problems, use of cannabis and use of other illicit drugs. Initially, covariates also included socio-economic position, domestic violence toward partner or sibling, psychological abuse and depressive symptoms. However, these were not associated with non-drinking and therefore excluded. Country of birth was classified as Sweden (student and both of their parents were born in Sweden), Europe (student or one of their parents was born elsewhere in Europe or North America), or Outside Europe (student or one of their parents was born outside Europe or North America). Family substance problems were coded if the student rated one family member as having a problem with substance use. Conflicts between parents and physical abuse were coded if present. More detailed information on family substance problems, conflicts between parents and physical abuse has been published elsewhere.^16^ Number of friends was measured as the sum of two items reflecting friends in school and in the neighbourhood, respectively, with a four-category scale from no close friends to three or more close friends. Status among friends was measured as the sum of two items for which the student rated his/her social status among classmates and among other friends, respectively, on a scale from zero to six. Psychosomatic problems were measured as the sum of nine items on how often the student suffered from symptoms, such as headache or stomach ache, rated on a five-category scale from never to always. More detailed information on psychosomatic problems has been published elsewhere.^17^ Sleeping problems were measured with a modified version of the Karolinska Sleep Questionnaire,^18^ as the sum of eight items, for instance, on difficulties waking up and insufficient sleep during the past 3 months, rated on a six-category scale from never to five times per week or more often. More detailed information on sleeping problems has been published elsewhere.^19^ Cannabis use and use of other illicit drugs were measured with one question each regarding how often the student had used such substances, rated on a seven-category scale from never to more than 50 times, dichotomized into no or yes.

#### Non-drinking alcohol

Alcohol consumption was measured with a version of the AUDIT-C questionnaire modified for adolescents.^20^ Responders were classified as non-drinkers if they had not consumed alcohol in the past 12 months.

#### Internet activities

Internet activities included total computer time, time spent on social media/chatting and time spent using computer games. Total computer time was measured with one item on the total number of hours per day spent on computers (not school related). Social media/chatting was measured with one item on how many hours per day on a five-category scale (not using computers, <1, 1–2, 2–5 and >5 hours) the student usually spent chatting on the internet/social media sites. Computer gaming was measured with a summarized score from two items on how many hours per day on a five-category scale (not using computers, <1, 1–2, 2–5 and >5 hours) the student usually played online computer games or other games on the internet on weekdays and on weekends, respectively. More detailed information on computer gaming has been published elsewhere.^21^

### Statistical analyses

First, in order to identify relevant covariates, bivariate logistic regression analyses were conducted calculating OR and 95% CI with non-drinking (no/yes) as the dependent variable (not shown in tables). Second, the distributions of internet activities and non-drinking from 2008 to 2012 were calculated separately for boys and girls. Third, bivariate associations between internet and non-drinking were calculated separately for boys and girls (not shown in tables), to evaluate whether associations differed. Since associations were similar for boys and girls, subsequent analyses were conducted on the total sample. Fourth, multivariate logistic regression analyses were conducted calculating OR and 95% CI with non-drinking (no/yes) as dependent variable and internet activities as independent variables adjusted for covariates. Analyses were conducted for each survey year in three models, where the first model included total computer time, the second model included internet activities (social media/chatting and computer gaming) and the third model divided computer gaming into weekdays and weekends. Fifth, in order to capture whether computer gaming on weekdays and/or on weekends was associated with non-drinking, computer gaming was divided into playing on weekdays only, on weekends only, and on both weekdays and weekends, in order to assess which of these activities, if any, was associated with non-drinking. Finally, differences between survey years and between boys and girls were evaluated as interaction effects. All analyses were conducted in SPSS version 22.0.

## Results

The distribution of internet activities and non-drinkers among students in 9th grade during the period 2008–2012 is shown in table 1. The proportion of non-drinkers increased from 42.8 to 52.2% for boys and from 36.1 to 46.0% for girls. Similarly, the proportion that used computers ≥2 hours/day also increased for both boys and girls, as well as the proportions that chatted and played computer games ≥2 hours/day, except for girls playing computer games. Noteworthy, the proportion of boys that used social media/chatted ≥2 hours/day increased substantially from 18.0 to 72.2%. Detailed analyses showed in particular an increase from 5.6 to 44.8% among boys that chatted/used social media ≥5 hours/day.

**Table 1:** Distribution of non-drinking and internet activities in percentages (frequency) presented separately for boys and girls for each survey year

	Boys			Girls		
Year	2008	2010	2012	2008	2010	2012
n size	1291	1179	1028	1314	1260	1017
Alcohol						
Non-drinking	42.8%	49.8%	52.2%	36.1%	39.2%	46.0%
	(540)	(571)	(519)	(469)	(488)	(460)
Internet activities						
Total computer time						
≥ 2 hours/day	59.8%	59.9%	69.6%	42.1%	42.9%	57.6%
	(754)	(680)	(649)	(543)	(529)	(556)
Social media/chatting						
≥2 hours/day (chatting)	19.4%	18.0%	72.2%	31.3%	28.7%	46.9%
	(236)	(202)	(712)	(388)	(345)	(462)
Computer games						
≥2 hours/weekday	44.4%	47.6%	66.6%	8.7%	13.8%	10.1%
	(533)	(519)	(645)	(104)	(158)	(98)
≥ 2 hours/weekend day	52.5%	60.4%	71.1%	11.7%	13.6%	11.5%
	(630)	(672)	(692)	(140)	(157)	(113)

Note. The *n* sizes at the top of the table include non-responders for each variable. Thus, summarizing the frequencies for all variables do not add up to the n-size for each survey year since non-responders were excluded when percentage was calculated. The rates of non-responders varied among boys from 2.3% (*n* = 30) for non-drinking in 2008 to 9.2% (*n* = 95) for total computer time in 2012. Among girls, the rates of non-responders varied from 1.2% for non-drinking in 2008 to 9.3% (*n* = 117) for computer games on weekdays in 2010.

Internet activities were associated with non-drinking in bivariate models (not shown in tables). The total computer time decreased the probability of non-drinking among both boys (OR = 0.89, CI = 0.83–0.96) and girls (OR = 0.83, CI = 0.76–0.89). Social media/chatting decreased the probability of non-drinking among both boys (OR = 0.72, CI = 0.68–0.77) and girls (OR = 0.66, CI = 0.61–0.70), whereas computer gaming increased the probability of non-drinking among both boys (OR = 1.03, CI = 1.00–1.06) and girls (OR = 1.05, CI = 1.02–1.09). Since associations were similar for boys and girls, subsequent analyses were conducted on a gender-pooled sample.

Next, three multivariate models shown in table 2 of associations between internet activities and non-drinking were calculated, adjusting for covariates. The first model included the total number of hours that adolescents spent on computers per day. When adjusted for covariates, the total number of computer hours was not associated with non-drinking regardless of survey year (Model 1). The second model separated different types of internet activities, such as social media/chatting and computer gaming. Social media/chatting was robustly associated with a decreased probability of non-drinking across the three survey years, whereas computer gaming was associated with an increased probability of non-drinking, but only in 2012 (Model 2). The third model separated computer gaming on weekdays from weekends. Computer gaming on weekends, but not on weekdays, was associated with an increased probability of non-drinking from year 2010 (Model 3).

**Table 2:** Multivariate associations between non-drinking as the dependent variable and different types of internet activities

	Year 2008	Year 2010	Year 2012
	OR (CI)	OR (CI)	OR (CI)
Model 1			
Gender (boys as reference)	0.85 (0.70–1.03)	0.68 (0.66–0.83)	0.70 (0.56–0.88)
Total computer hours	0.91 (0.83–0.99)	0.93 (0.84–1.03)	1.02 (0.90–1.15)
Model 2 (hours/day)			
Gender (boys as reference)	1.17 (0.92–1.49)	0.88 (0.69–1.13)	0.91 (0.68–1.23)
Social media/chatting	0.83 (0.79–0.86)	0.82 (0.77–0.86)	0.83 (0.77–0.88)
Computer games	1.03 (0.99–1.07)	1.00 (0.96–1.05)	1.05 (1.00–1.10)
Model 3 (hours/day)			
Gender (boys as reference)	1.19 (0.93–1.51)	0.91 (0.70–1.17)	0.96 (0.71–1.29)
Social media/chatting	0.83 (0.79–0.86)	0.81 (0.77–0.86)	0.82 (0.76–0.88)
Computer games weekdays	0.94 (0.80–1.10)	0.87 (0.77–1.00)	0.96 (0.84–1.11)
Computer games weekends	1.13 (0.98–1.30)	1.13 (1.00–1.28)	1.15 (1.00–1.32)

Note. Model 1 includes total computer time, Model 2 includes social media/chatting and computer games, and Model 3 includes computer games divided into weekdays and weekends. The models are adjusted for covariates including country of birth, family substance problems, conflicts between parents, physical abuse, number of friends, status among friends, psychosomatic problems, sleeping problems, use of cannabis and use of other drugs.

However, adolescents can engage in computer gaming on both weekdays and weekends. Thus, we divided them into four groups: <2 hours/day on weekdays and weekends (boys *n* = 35.4%, girls *n* = 84.3%); ≥2 hours/day on weekdays only (boys *n* = 4.5%, girls *n* = 3.5%); ≥2 hours on weekends only (boys *n* = 13.4%, girls *n* = 4.9%); and ≥2 hours on both weekdays and weekends (boys *n* = 46.7%, girls *n* = 7.3%). Associations between these groups and non-drinking, adjusted for covariates, are shown in table 3. Playing computer games ≥2 hours on weekends only (OR = 1.74, CI = 1.13–2.69) or both on weekdays and weekends (OR = 1.82, CI = 1.31–2.54) were associated with an increased probability of non-drinking, but only in year 2012. These two groups constituted 60.1% of boys and 12.2% of girls in 2012 (not shown in tables).

**Table 3:** Multivariate associations between non-drinking as the dependent variable and computer games when divided into weekdays only, weekends only and both weekdays and weekends

	Year 2008	Year 2010	Year 2012
	OR (CI)	OR (CI)	OR (CI)
Gender (boys as reference)	0.98 (0.78–1.23)	0.81 (0.64–1.03)	0.84 (0.64–1.12)
Social media/chatting	0.57 (0.45–0.73)	0.57 (0.44–0.72)	0.53 (0.41–0.67)
Computer games weekdays only	0.54 (0.27–1.08)	0.78 (0.48–1.28)	1.14 (0.66–1.95)
Computer games weekends only	1.07 (0.76–1.53)	1.14 (0.83–1.57)	1.74 (1.13–2.69)
Computer games weekdays and weekends	1.23 (0.95–1.58)	1.12 (0.86–1.46)	1.82 (1.31–2.54)

Note. Chatting ≥ 2 hours.day. Playing computer games is divided into four groups: ≥2 hours/day on weekdays or weekends; ≥2 hours/day on weekdays only, ≥ 2 hours on weekends only, or ≥ 2 hours on both weekdays and weekends. The models are adjusted for covariates including country of birth, family substance problems, conflicts between parents, physical abuse, number of friends, status among friends, psychosomatic problems, sleeping problems, use of cannabis and use of other drugs.

Possible differences in the associations between internet activities and non-drinking between survey years and between genders were evaluated as interaction effects and are shown in table 4. Only one interaction between gender and chatting was significant, indicating a stronger negative association between chatting and non-drinking for girls than for boys.

**Table 4:** Differences between survey years and gender differences in the associations between internet activities and non-drinking measured with interaction effects

	OR (CI)
Effect of survey year	
Social media/chatting × survey year	1.00 (0.97–1.03)
Computer games weekdays × survey year	1.03 (0.98–1.09)
Computer games weekend × survey year	1.01 (0.97–1.06)
Gender differences	
Social media/chatting × gender (girl)	0.86 (0.78–0.95)
Computer games weekdays × gender (girl)	1.12 (0.95–1.32)
Computer games weekend × gender (girl)	0.96 (0.82–1.11)

Note. The interaction effects of survey year are adjusted for gender and all covariates. The interaction effects of gender are adjusted for survey year and all covariates. Main effects were also included in the models. Adjusted covariates included country of birth, family substance problems, conflicts between parents, physical abuse, number of friends, status among friends, psychosomatic problems, sleeping problems, use of cannabis and use of other drugs.

## Discussion

The present study examined the suggestion that adolescents do not drink alcohol since they spend more time on the internet in domestic environments that more easily can be monitored by parents,^7^^,^^8^ in a population-based sample of 9th graders (15–16 years) in Sweden.

First, the present study does not confirm that time spent on the internet is related to non-drinking, since the total time spent on computers was associated with a decreased probability of non-drinking when covariates were adjusted for. On the other hand, when disentangled, playing computer games was associated with an increased probability of non-drinking, when adjusted for covariates, in the 2012 survey only. The finding makes sense, as the largest increase of 9th graders who played computer games ≥2 hours/day occurred between the 2010 and the 2012 surveys, in particular for boys. Further, previous studies of non-drinking among adolescent computer gamers have yielded mixed findings.^11^^,^^12^^,^^15^ However, the present study extends these findings by showing that the increased probability of non-drinking among computer gamers is restricted to those who play on weekends, or on weekdays and weekends. This makes sense, as adolescents often drink alcohol on weekends rather than on weekdays.^22^

Second, although computer gaming especially on weekends was associated with non-drinking in 2012, the effect sizes were relatively small. On the other hand, the proportion of adolescent boys among whom computer gaming was associated with non-drinking was large, encompassing 60.1% of all boys in the 2012 survey. Like the prevention paradox, where the group with low or moderate risk can be responsible for the majority of related problems due to its size,^23^ the effect size of computer gaming is small, but has the potential to impact on the majority of boys. Further, no differences in the associations between internet activities and non-drinking during the 4-year period were found, which implies that internet activities have a limited impact on the increased trend toward non-drinking that occurred in the past 15 years. However, this finding should be interpreted with caution, since the study covered a limited time period of 4 years.

Third, the time spent on social media/chatting was robustly associated with decreased odds of non-drinking across the three survey years, with a stronger association among girls than boys. This finding is in line with others suggesting that this association probably is driven by displayed alcohol references on these sites,^9^^,^^10^ and online alcohol marketing.^24^^,^^25^ Thus, online chatting/social media platforms may constitute a forum where adolescents are exposed for positive alcohol preferences from peers and alcohol marketing without parental scrutiny, which possible reinforces peer communication at the expense of communication with parents,^26^ and weaken the protective effect of parental monitoring.^27^ Further, the substantial increase of boys that use social media/chat ≥2 hours/day from 18.0% in 2010 to 72.2% in 2012 is odd given that adolescent girls in Sweden use social media more than boys.^28^ However, the daily use of internet in smartphones dramatically increased from 5% in 2010 to 38% in 2012,^29^ and it is possible that adolescent boys were quicker to adopt to the new technology.

### Advantages and limitations

Advantages of the present study include the use of three large population-based samples of all 9th graders (15–16 years) in a mid-sized county in Sweden, allowing us to study differences between survey years and gender differences. Another strength is that different types of internet activities were addressed. Limitations of the study include the cross-sectional design, precluding any interpretations about causality, and that data were not available for non-responders, making it difficult to interpret how non-responders influenced the investigated associations. Further, surveys distributed in 2008–2012 were used, which is a limited time period of 4 years. Thus, valid conclusions about whether computer gaming has contributed to the increased trend toward non-drinking that has occurred since 2000 cannot be done. Furthermore, the present study do not take other competing trends into consideration such as alcohol policy changes, changes in parental norms of offspring drinking,^30^ increase of academic demands,^31^ global changes such as the global economic recession that occurred in the late 2000s, or if adolescents use illicit drugs instead of alcohol. Regarding the latter, however, the proportion of non-drinking 9th graders that use illicit drugs was lower than 2.5% from 1995 to 2012 (submitted manuscript).

## Conclusions

In general, total computer time is not associated with non-drinking among adolescents. Although computer gaming in particular during weekends is associated with an increased probability for non-drinking in 2012, the effect size is relatively small and therefore not likely to contribute more than marginally to the increased trend toward non-drinking. On the other hand, the proportion of boys that benefits from this small effect size is large. Further, chatting/social media use was associated with a decreased probability of non-drinking. Thus, these online platforms may constitute forums where adolescents are influenced by positive alcohol references and online alcohol marketing, without parental scrutiny.

## Funding

This study was funded by grants from the Swedish Research Council for Health, Working Life and Welfare (#2015-00857) and the Systembolaget’s Research Council on Alcohol (#FO2016-0073).

*Conflicts of interest*: The present study received funding from Systembolaget’s Research Council on alcohol (#FO2016-0073). While Systembolaget is the national alcohol monopoly company in Sweden, the funders played no role in the study design, analysis or interpretation, or in the decision of where to submit the paper for publication.

**Key points**

- The present study does not confirm the suggestion that adolescents do not drink alcohol because they spend more time on internet.
- Computer gaming in particular during weekends was associated with non-drinking. Although a small effect size, the majority of boys benefits from this small effect size.
- The time spent on social media/chatting was robustly associated with decreased odds of non-drinking across the three survey years, with a stronger association among girls than aming boys.
